# Comparison of Patterns and Demographics of Isolated Traumatic Mandibular Fracture Between Incarcerated and General Populations

**DOI:** 10.7759/cureus.60458

**Published:** 2024-05-16

**Authors:** Thuy-My Le, Carlo LaGatta, James Lelis, Cameron C Neeki, Elvin Chiang, Arianna S Neeki, Amy Choi, Ashley Choi, Fanglong Dong, Michael M Neeki

**Affiliations:** 1 Emergency Medicine, Arrowhead Regional Medical Center, Colton, USA; 2 Oral Maxillofacial Surgery, Loma Linda University Medical Center, Loma Linda, USA; 3 Medicine, California University of Science and Medicine, Colton, USA

**Keywords:** penetrating trauma, blunt trauma, mortality, emergency department, mandible fracture, maxillofacial trauma

## Abstract

Introduction

The management of maxillofacial trauma can be challenging in different unique clinical presentations. While maxillofacial fractures vary in location based on the mechanism of injury, the mandibular fracture is noted to be one of the most common facial fractures. The objective of this study was to explore the differences in injury patterns, outcomes, and demographics of isolated traumatic mandibular fractures between incarcerated and general populations.

Methods

This retrospective study analyzed consecutive patients presenting for trauma care from January 1, 2010, to December 31, 2020, at the Arrowhead Regional Medical Center (ARMC). Patients 18 years and older were included in this study. Patients diagnosed with mandibular fracture as the primary diagnosis at admission and discharge were identified using the International Classification of Disease, Ninth and Tenth Revision (ICD-9, ICD-10) Code. Patient demographics were extracted from their electronic medical records and included race, marital status, and insurance status.

Results

A total of 1080 patients with confirmed mandibular fractures were included in the final analysis. Among these patients, 87.5% (n=945) were males, 40% (n=432) of the patients were Hispanic, and the average age was 31.55 years old. The most common mechanism of injury was blunt trauma secondary to assault. Compared to the general population with mandibular fracture, the incarcerated patients with mandibular fracture were more likely to be males (96.1% vs 86.1% for incarcerated population vs. general population respectively, p=0.0005). No other variables were statistically different between these two groups.

Conclusion

The evidence from this study suggests that the patterns, outcomes, and demographics of mandibular fracture in both incarcerated and general populations are similar.

## Introduction

Maxillofacial trauma and in particular traumatic mandibular fractures can be challenging in different populations with their unique presentations [1]. While the type of maxillofacial fractures is determined by the mechanism of injury and force vectors, the mandible is considered one of the most common fracture sites [1, 2]. Mandibular fractures can result from a multitude of trauma mechanisms such as motor vehicle accidents, sports injuries, or physical assaults [1].

Moreover, mandibular fractures deserve special attention as management can greatly vary, ranging from requiring no treatment to surgical intervention. This may depend on the number of sites involved, the degree of displacement, and the presence of other facial fractures/injuries [3]. For example, treatment of an anterior mandible fracture that involves symphysis and parasymphysis is relatively simple as it may only require closed reduction by applying stainless steel wires. In contrast, the treatment of a condylar fracture may be more complicated as the management approach is not well agreed upon in the literature [3].

In order to efficiently identify and manage mandible fractures, it is important to define the demographics and patterns that surround this particular injury. There are several variables that contribute to the prevalence of mandible fractures within a population such as location, population size, socio-economic status, regional government, and culture [3]. Panesar and Susarla have identified various demographic and socioeconomic factors associated with mandibular trauma in the general population [3], but the literature has been scant regarding the incarcerated population [4]. This population is of particular interest as the incarcerated population is more likely to experience facial injuries than the general population due to the increased level of violence and assault.

This study aims to explore the differences in injury patterns, outcomes, and demographics of isolated traumatic mandibular fractures between incarcerated and general populations. The findings may improve healthcare for this marginalized population.

## Materials and methods

This retrospective study analyzed consecutive patients presenting for trauma care from January 1, 2010, to December 31, 2020, at the Arrowhead Regional Medical Center (ARMC). ARMC is a 456-bed acute care teaching facility and an American College of Surgeons certified level I trauma center in San Bernardino County, California. The ARMC emergency department (ED) is one of the busiest in the state of California with more than 100,000 visits and over 3,000 adult trauma cases annually [5].

Patients 18 years and older were included in this study. Patients diagnosed with mandibular fracture as the primary diagnosis at admission and discharge were identified using the International Classification of Disease, Ninth and Tenth Revision (ICD-9, ICD-10) Code. Patient demographics were extracted from their electronic medical records and included race, marital status, and insurance status. This study was approved by the institutional review board at ARMC with the approval number 20-39. Informed consent was waived, and data were reported in an aggregated format. No patients were identified in this article.

Two groups were created to fulfill the study objective. The first group was the incarcerated patient population and the second group was the general population. The incarcerated patient population was mainly from regional prisons managed by local, state, and federal authorities. In addition, the trauma to the patients originated while housed inside the facilities. The general population includes those who were transported by ambulance, self-arrival, or other modes of transportation. These patients were involved in various traumatic injuries which included but were not limited to motor vehicle accidents, assaults, and other activities.

All statistical analyses were conducted using the SAS software for Windows 9.4 (SAS Institute, Cary, USA). Descriptive statistics were presented as mean and standard deviations (SD) for continuous variables, along with frequencies and proportions for categorical variables. Chi-square Crosstab tests were conducted to assess the association between categorical variables and groups (general vs incarcerated) separately. Independent t-tests were conducted to assess whether the continuous variables were different between groups (general vs incarcerated). All statistical tests were two-sided. A p-value <0.05 was considered statistically significant.

## Results

**Figure 1:**
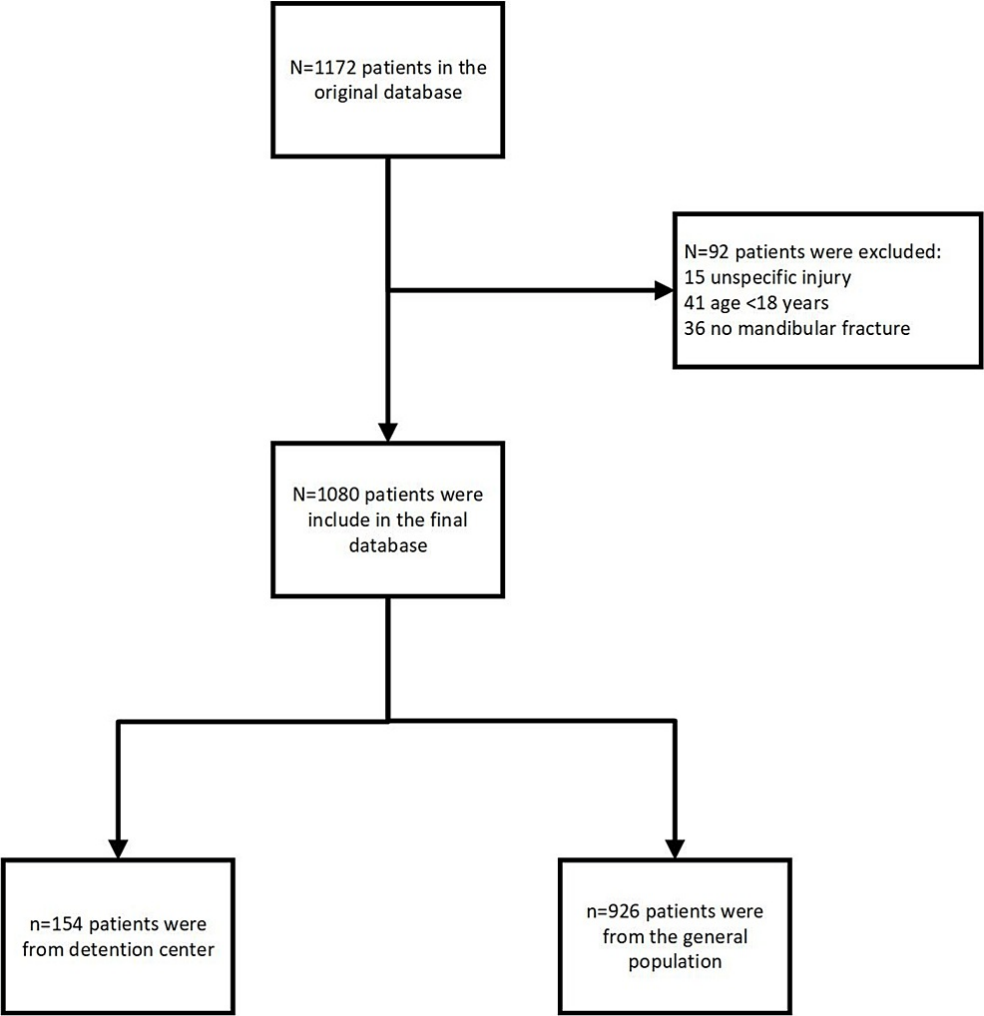
Patient flow chart

Originally, 1172 patients were included in the original database. After excluding 92 patients, 1080 patients who sustained mandibular fractures were included in the final analysis. Figure 1 presents the patient flow chart.

Among the 1080 patients, the majority 87.5% (n=945) of the patients were males, 40% (n=432) of patients were Hispanic, 56.3% (n=608) had Medi-Cal insurance, and the average age was 31.55 years old. The most common mechanism of injury was assault at 70.9% (n=766), and 98.4% (n=1063) of patients sustained blunt trauma. 15.4% (n=166) of patients had positive urine drug screen (UDS), and 14.4% (n=156) had serum alcohol above legal limits. The incidence of mandibular fracture was evenly distributed over 12 months. The most common fracture location was angle (25.7%, n=278), and single location injury accounted for more than half (54.2%, n=585) of the injuries. More detailed demographic summaries can be found in Table 1.

**Table 1:** Comparison of variables between patients at the detention center and the general population. All variables except age and length of stay were presented as frequencies and proportions. Age was presented as mean ± standard deviations. Length of stay was presented as median with first and third quartile inside the parenthesis. MVA: motor vehicle accident; MCC: motorcycle crash; GSW: gunshot wound; ATV: all-terrain vehicle

	Overall (N=1080)	Detention Center (n=154)	General population (n=926)	P-value
Male Gender	945 (87.5%)	148 (96.1%)	797 (86.1%)	0.0005
Ethnicity				0.0775
African American	295 (27.3%)	49 (31.8%)	246 (26.6%)	
Asian	14 (1.3%)	1 (0.7%)	13 (1.4%)	
Caucasian	339 (31.4%)	56 (36.4%)	283 (30.6%)	
Hispanic	432 (40%)	48 (31.2%)	384 (41.5%)	
Alcohol above legal limits	156 (14.4%)	8 (5.2%)	148 (16%)	0.0004
Urine drug screening positive	166 (15.4%)	21 (13.6%)	145 (15.7%)	0.5194
Insurance				< 0.0001
No insurance	386 (35.7%)	154 (100%)	232 (25.1%)	
Medi-Cal	608 (56.3%)	0	608 (65.7%)	
Private Insurance	86 (8%)	0	86 (9.3%)	
Mechanism of injury				N/A
Assault	766 (70.9%)	119 (77.3%)	647 (69.9%)	
Fall	162 (15%)	22 (14.3%)	140 (15.1%)	
MVA	43 (4%)	1 (0.7%)	42 (4.5%)	
MCC	35 (3.2%)	0 (0%)	35 (3.8%)	
Sport injury	34 (3.2%)	3 (2%)	31 (3.4%)	
GSW	15 (1.4%)	3 (2%)	12 (1.3%)	
Seizure	13 (1.2%)	4 (2.6%)	9 (1%)	
ATV	12 (1.1%)	2 (1.3%)	10 (1.1%)	
Admission Month				N/A
January	84 (7.8%)	11 (7.1%)	73 (7.9%)	
February	51 (4.7%)	7 (4.6%)	44 (4.8%)	
March	87 (8.1%)	8 (5.2%)	79 (8.5%)	
April	104 (9.6%)	15 (9.7%)	89 (9.6%)	
May	91 (8.4%)	13 (8.4%)	78 (8.4%)	
June	105 (9.7%)	20 (13%)	85 (9.2%)	
July	93 (8.6%)	11 (7.1%)	82 (8.9%)	
August	96 (8.9%)	8 (5.2%)	88 (9.5%)	
September	97 (9%)	16 (10.4%)	81 (8.8%)	
October	93 (8.6%)	15 (9.7%)	78 (8.4%)	
November	101 (9.4%)	17 (11%)	84 (9.1%)	
December	78 (7.2%)	13 (8.4%)	65 (7%)	
Fracture location				N/A
Involving 1 Location				
Angle	278 (25.7%)	41 (26.6%)	237 (25.6%)	
Symphysis	78 (7.2%)	17 (11%)	61 (6.6%)	
Body	94 (8.7%)	14 (9.1%)	80 (8.6%)	
Condyle	58 (5.4%)	7 (4.6%)	51 (5.5%)	
Subcondylar	36 (3.3%)	5 (3.3%)	31 (3.4%)	
Ramus	31 (2.9%)	4 (2.6%)	27 (2.9%)	
Involving 2 Locations				
Angle & Body	175 (16.2%)	30 (19.5%)	145 (15.7%)	
Angle & Symphysis	129 (11.9%)	10 (6.5%)	119 (12.9%)	
Subcondylar & Symphysis	59 (5.5%)	6 (3.9%)	53 (5.7%)	
Body & Condyle	23 (2.1%)	6 (3.9%)	17 (1.8%)	
Body & Ramus	23 (2.1%)	3 (2%)	20 (2.2%)	
Body & Subcondylar	25 (2.3%)	3 (2%)	22 (2.4%)	
Condyle & Symphysis	24 (2.2%)	2 (1.3%)	22 (2.4%)	
Subcondylar & Ramus	2 (0.2%)	1 (0.7%)	1 (0.1%)	
Body & Symphysis	13 (1.2%)	1 (0.7%)	12 (1.3%)	
Angle & Condyle	3 (0.3%)	0	3 (0.3%)	
Angle & Ramus	1 (0.1%)	0	1 (0.1%)	
Angle & Subcondylar	2 (0.2%)	0	2 (0.2%)	
Ramus & Symphysis	13 (1.2%)	0	13 (1.4%)	
Involving 3 or more locations				
Condyle & Ramus & Symphysis	2 (0.2%)	2 (1.3%)	0	
Angle & Body & Condyle	2 (0.2%)	1 (0.7%)	1 (0.1%)	
Angle & Body & Ramus	3 (0.3%)	1 (0.7%)	2 (0.2%)	
Angle & Condyle & Symphysis	1 (0.1%)	0	1 (0.1%)	
Angle & subcondylar & ramus	1 (0.1%)	0	1 (0.1%)	
Angle & Subcondylar & Symphysis	1 (0.1%)	0	1 (0.1%)	
Angle & Body & Condyle & Symphysis	1 (0.1%)	0	1 (0.1%)	
Angle & Body & Subcondylar	2 (0.2%)	0	2 (0.2%)	
Blunt vs penetrating				0.6872
Blunt	1063 (98.4%)	151 (98.1%)	912 (98.5%)	
Penetrating	17 (1.6%)	3 (2%)	14 (1.5%)	
Single vs multiple injury				0.2946
Single location injury	585 (54.2%)	88 (57.1%)	487 (52.6%)	
Multi location injury	495 (45.8%)	66 (42.9%)	439 (47.4%)	
Length of stay				0.3666
0 day	942 (87.2%)	133 (86.4%)	809 (87.4%)	
1 day	102 (9.4%)	18 (11.7%)	84 (9.1%)	
2+ days	36 (3.3%)	3 (2%)	33 (3.6%)	
Number of mandibular fracture				0.1359
1	575 (53.2%)	88 (57.1%)	487 (52.6%)	
2	492 (45.6%)	62 (40.3%)	430 (46.4%)	
3	12 (1.1%)	4 (2.6%)	8 (0.9%)	
4	1 (0.1%)	0	1 (0.1%)	
Age	31.55 ± 12.26	33.29 ± 31.5	31.26 ± 27	0.0570
Length of stay in hospital	0 (0,0)	0 (0,0)	0 (0,0)	0.7305

A comparison between patients from the detention centers and the general population was conducted and the analysis results were also presented in Table 1. Compared to the general population who sustained a mandibular fracture, the incarcerated patients were more likely to be males (96.1% vs 86.1%, p=0.0005), more likely to have no insurance (100% vs 25.1%, p<0.0001), and less likely to be under the influence of alcohol (5.2% vs 16%, p=0.0004). No other statistically significant differences were noted in other variables between the incarcerated and general population.

## Discussion

The current study noted that the mean age of mandible fractures among the incarcerated versus the general population is similar. Regardless of population, maxillofacial fractures have been noted to occur more frequently in younger individuals in the 21-30 year-old age group [6-8]. This can be attributed to their higher inclination to be involved in physical activities, reckless driving, alcohol abuse, interpersonal violence, and participation in different sports activities [9]. In the incarcerated population, a younger demographic for mandibular fractures likely exists due to a proclivity for violence in general, but especially among those who are in custody [10].

As expected, the male-to-female ratio among the incarcerated population (25:1) was significantly greater than that of the general population (6:1). This statistical difference directly correlates with the higher proportion of incarcerated men than women [11]. In comparison with women, men are also more likely to commit more serious crimes and be repeat offenders due to the suggested innate gender differences in psychological, biological, and sociological factors [11-13].

Location of injury

The result of this study revealed a higher frequency of closed single-location mandibular fractures. Ellis et al. noted a similar higher prevalence of single-location mandibular fractures and the association with low-velocity assault injuries seen in detention centers [14]. Intuitively, low-impact trauma from an assault to a small area of the face would less likely have the kinetic energy and force required to cause multiple fracture sites as compared to a high-impact mechanism such as a motor vehicle accident [15].

When comparing the incarcerated population to the general population, there was no significant difference in the most common type of fracture. The mandibular angle was the most common fracture site noted in both the incarcerated (26.6%) and the general population (25.6%). Morris and colleagues noted an association between the mechanism of injury and type of fracture [15]. More specifically, angle was the most common site of fracture for low-velocity blunt trauma; condylar was the most common site of injury for high-velocity blunt trauma; symphysis was the common site of fracture for low-velocity penetrating trauma, and body fracture was the most common site of fracture for high-velocity penetrating trauma [15]. In a separate study where higher velocity mechanisms were the most common among their study population, Alharbi and colleagues identified the body of the mandible as the most frequent site of fracture followed by mandibular angle fractures [6]. In the same study, the investigators suggested that the mandibular angle is located in a more protected area and is therefore less likely to be affected by general trauma in comparison to trauma in detention centers [6]. 

This study along with prior literature continues to present evidence on how the mechanism of the injuries greatly influences the location of the fracture despite variation in the population. While the mandibular angle is the most common type of fracture identified among incarcerated patients who predominantly encounter low-velocity assault, a future study within this population would be worthwhile to investigate if the location of mandibular fractures is further influenced by the type of weapons used in an assault (i.e., fist versus other blunt objects).

Frequency of combination fractures

Although single-location fractures are more common, when combination fractures manifest, this study revealed a higher rate of angle + body fractures in the incarcerated population in comparison to the general population. This may be attributed to assault as the main cause of mandibular fractures in incarcerated patients as opposed to other low-velocity mechanisms experienced by the general population such as falls. Studies have reported that blows to the face and jaw are common in altercations, leading to a higher rate of angle and body combination fractures [16]. While mandibular body fracture management is contingent on the severity of the injury, the need for operative management may increase with a compounded angle fracture [17]. Moreover, the treatment of mandibular angle fractures is itself complicated without a clear consensus on the best method of treatment. Currently, it is still contested whether the optimal treatment for angle fractures is a rigid fixation with intraoperative maxillomandibular fixation versus non-compression miniplates [18]. There may be other cultural factors that could contribute to the higher incidence of combination fractures. The severity of injury may well be affected by the higher rate of substance use in the incarcerated population, which increases the risk of injury due to decreased coordination and judgment during an altercation [19, 20]. There could be an element of delayed presentation in the incarcerated population too due to the inability to access adequate healthcare in correctional facilities and/or a culture of toughness [21]. Rather than presenting to the hospital after the first facial injury, incarcerated patients are more likely to present only at times when pain or decreased functionality due to compounded injuries compel them to aggressively seek medical attention.

Furthermore, among combination fractures, this study also reports a higher proportion of angle + symphysis fractures in the general population in comparison to the incarcerated population. This noted difference is likely due to the variations in the primary mechanisms of mandibular fractures for each population. In the general population, higher impact mechanisms of injury (e.g., motor vehicle accidents) are more common [9]. Angle and symphysis combination fractures require significant force of impact to be distributed across the mandible [9]. Of note, this level of kinetic force is generally not usually possible to be applied physically by one inmate to another’s face.

Inpatient versus outpatient management

The result of this study revealed that after the initial trauma evaluation, all of the patients were treated in an outpatient setting. This is in stark contrast to Henning and colleagues who found among their population of incarcerated patients with mandibular fractures - 96% required admission, with 84% of those patients undergoing surgery during the admission [22]. Notably, mandible fractures were the most common facial fracture seen in that study [22]. The key aspect of that study is the fact that the standard of care for managing mandibular fractures may have changed over the past years. In addition, it is known that various hospitals and correctional institutions have different protocols, policies, and levels of access to oral and maxillofacial specialists which may change the standard of care in that particular institution.

Based on current results, treating isolated mandibular fractures on an outpatient basis may be a safe, feasible, and institutionally cost-effective approach [23]. Outpatient management has been shown to reduce hospital costs by reducing the length of hospital stay and reducing patient discomfort and inconvenience when compared to inpatient treatment [23]. Elek and colleagues also reported no significant difference in the outcomes between patients treated on an outpatient basis and those treated inpatients for isolated open mandibular fractures [23]. In fact, outpatient management mitigates the risk of hospital-acquired infections and complications associated with prolonged hospital stays, which would be further complicated in an incarcerated patient who has limited access to medical care [24]. Given that most of the mandibular fractures experienced by incarcerated patients are single-site fractures as noted in this study, admission is not likely required for management.

Substance abuse

This study revealed that the proportion of alcohol use in the incarcerated population was significantly lower than that in the general population. This finding is contrary to previous research that suggests incarcerated individuals have higher rates of alcohol use than the general population [22]. In contrast, incarcerated individuals have higher rates of having a history of substance use disorders which are often perpetuated in jail [23]. The difference in the results between the current study and previously published literature may likely be attributed to the fact that those who were arrested were jail-checked prior to booking and therefore had a lower serum alcohol level upon assessment. In addition, the majority of the population in this study came from the local county jails that have strict isolation and do not usually have access to alcohol. 

The investigating team in this study was interested in exploring this component of mandibular fractures because the literature has shown that a history of tobacco and alcohol use is correlated with the development of infection, nonunion, and related complications after the repair of mandibular fractures. Whereas demographics, fracture site, lag time to repair, and even use of antibiotics had no correlation [24]. This further supports outpatient management of mandible fractures of incarcerated patients, as it can be derived that they are less prone to complications due to the lack of alcohol use. Contrastingly, there was no statistically significant difference between the proportion of positive urine drug screenings in the incarcerated group (13.6%) and the general group (15.7%).

Limitations

This study primarily focused on the comparison of patterns and demographics of mandible fractures of incarcerated patients versus the general population. Due to the sensitive nature of the study population, not all information was available. However, the research team made an effort to retrieve all relevant information. Secondly, all incarcerated populations were managed in a single level-I trauma center. Therefore, the findings in the current study may be limited beyond the current institution. Future research needs to also focus on variation of management, availability of services, and long-term outcomes of the target population undergoing surgical procedures.

## Conclusions

Incarcerated patients present unique challenges for healthcare providers based on variations in their local institutional policies. There is no statistically significant difference in patterns and demographics of mandibular fractures between incarcerated and generation populations. This study emphasizes that with a good specialty follow-up system in place, mandibular fractures of incarcerated patients can be appropriately managed in the outpatient setting.
